# Epilepsy in NF1: Epidemiologic, Genetic, and Clinical Features. A Monocentric Retrospective Study in a Cohort of 784 Patients

**DOI:** 10.3390/cancers13246336

**Published:** 2021-12-17

**Authors:** Ugo Sorrentino, Silvia Bellonzi, Chiara Mozzato, Valeria Brasson, Irene Toldo, Raffaele Parrozzani, Maurizio Clementi, Matteo Cassina, Eva Trevisson

**Affiliations:** 1 Clinical Genetics Unit, Department of Women’s and Children’s Health, University of Padova, 35128 Padua, Italy; chiara.mozzato@studenti.unipd.it (C.M.); valeria.brasson@studenti.unipd.it (V.B.); maurizio.clementi@unipd.it (M.C.); matteo.cassina@unipd.it (M.C.); 2 Pediatrics Complex Care Unit, Santa Maria della Misericordia Hospital, 45100 Rovigo, Italy; silvia.bellonzi@aulss5.veneto.it; 3 Pediatric Neurology Unit, Department of Women’s and Children’s Health, University Hospital of Padua, 35128 Padua, Italy; irene.toldo@unipd.it; 4 Department of Neuroscience-Ophthalmology, University of Padova, 35128 Padua, Italy; raffaele.parrozzani@unipd.it; 5 Institute of Pediatric Research IRP, “Fondazione Città della Speranza”, 35127 Padua, Italy

**Keywords:** neurofibromatosis 1, *NF1*, epilepsy, seizures, neurological comorbidities, cerebral vasculopathies, brain neoplasms, epidemiology

## Abstract

**Simple Summary:**

Neurofibromatosis type 1 is a relatively frequent neurocutaneous and tumor predisposition syndrome, which has been associated with a variety of neurological manifestations, including an increased incidence of seizures. Epilepsy in NF1 has been investigated over time by a number of studies, but most of these works were performed on small samples and often suffered from selection biases. Our study aimed to provide additional epidemiologic, clinical, and molecular data to the literature by retrospectively analyzing a large cohort of consecutive unselected patients affected with NF1. Such data may provide a better insight into the matter to other authors and clinicians alike.

**Abstract:**

An increased lifetime risk of epilepsy has been reported in neurofibromatosis type 1 (NF1) patients, ranging between 4% and 14%. To further analyze the correlation between NF1 and epilepsy, we retrospectively reviewed the epidemiologic, clinical, radiological, and molecular data of 784 unselected patients diagnosed with NF1 and referred to the neurofibromatosis outpatient clinics at the University Hospital of Padua. A crude prevalence of epilepsy of 4.7% was observed. In about 70% of cases, seizures arose in the context of neuroradiological findings, with the main predisposing factors being cerebral vasculopathies and hydrocephalus. In the absence of structural abnormalities, the prevalence of epilepsy was found to be 1.27%, which is approximately equal to the total prevalence in the general population. NF1 patients with seizures exhibit a higher incidence of intellectual disability and/or developmental delay, as well as of isolated learning disabilities. The comparison of causative *NF1* mutations between the two groups did not reveal a specific genotype–phenotype correlation. Our data refine the current knowledge on epileptological manifestations in NF1 patients, arguing against the hypothesis that specific mechanisms, inherent to neurofibromin cellular function, might determine an increased risk of epilepsy in this condition.

## 1. Introduction

Neurofibromatosis type 1 (NF1) is an autosomal dominant inherited neurocutaneous disease, with a prevalence ranging from 1/2000 to 1/5000 [1,2,3,4]. This condition is caused by heterozygous loss-of-function mutations of the *NF1* gene, which maps on chromosome 17q11.2 and encodes the tumor suppressor protein neurofibromin. NF1 shows marked allelic heterogeneity, with more than 3000 distinct pathogenic variants reported so far [5,6]. Although the *NF1* mutational spectrum is wide, only few genotype–phenotype correlations have been documented [7,8,9,10,11].

NF1 exhibits a multisystem involvement with heterogeneous clinical manifestations, including café-au-lait macules (CALMs), axillary and/or inguinal freckling, iris Lisch nodules and/or choroidal abnormalities, neurofibromas, optic pathway glioma (OPG), T2-weighted MRI scan unidentified bright objects (UBOs), and distinctive osseous lesions, such as sphenoid dysplasia and anterolateral bowing of the tibia [12,13]. In addition to these typical features, affected patients may present a variety of neurological manifestations, such as epilepsy, brain tumors other than OPG, hydrocephalus, cerebrovascular diseases (including moyamoya), migraine-like headache, intellectual disability, and other developmental abnormalities (i.e., learning disability, attention deficit-hyperactivity disorder—ADHD, and autism spectrum disorder—ASD) [14,15,16].

Specifically, the association between NF1 and an increased lifetime risk of epilepsy has been reported in different epidemiological studies, which estimated a lifetime prevalence of seizures in this condition ranging from 4% to 14% [17,18,19,20,21,22,23,24]. In the majority of cases, seizures have been described as characterized by a focal onset, but potentially becoming bilateral tonic-clonic seizures. However, no distinctive electroclinical patterns of seizures have been demonstrated in NF1 patients so far [17].

While the coexistence of NF1-related focal lesions, particularly brain tumors, vascular malformations, and cortical malformations, may account for approximatively half of the cases of seizures in NF1 patients [21,23], the causes behind the nonstructural cases remain to be determined [19,22,23]. The relationship between UBOs or OPGs and seizures has been consistently excluded [18,19,20,22], whilst the role of other brain abnormalities frequently described in NF1 patients, such as hydrocephalus, is still uncertain [24]. In addition to structural anomalies, epileptic NF1 patients display a higher incidence of other NF1-related neurological comorbidities, such as intellectual disability, psychomotor retardation, and learning difficulties, regardless of the severity of the seizures [25].

A number of etiopathological hypotheses have been proposed to explain the higher prevalence of seizures in NF1 patients. A reduction in the threshold for epileptic discharges has been postulated [22], as well as an increased release of downstream signals along the MAPK and mTOR pathways through hyperactivation of the Ras GTPase [22,23]; nevertheless, the actual impact of such mechanisms remains to be clarified. A series of promising NF1 animal models have also been developed, demonstrating altered GABA levels and an increased epileptogenicity and susceptibility to electric kindling [26,27,28,29]. Among the patients that do not exhibit cerebral structural abnormalities, a family history of epilepsy was observed in one study, suggesting a more complex heritability of the epileptic trait [23,24].

Our study aimed to further investigate the correlation between NF1 and epilepsy and to describe clinical features and possible comorbidities in a large cohort of consecutive unselected patients.

## 2. Results and Discussion

### 2.1. Epidemiologic Data

Our study was conducted on 784 consecutive NF1 patients (371 females, 413 males), first referred to our Neurofibromatoses Center because of a clinical diagnosis or suspicion of NF1 before 13 years of age. Their demographic characteristics are described in Table 1.

Epilepsy was reported in 37 out of 784 cases, with a crude prevalence of 4.72% (95% CI: 3.2%–6.2%) (24 males and 13 females). This datum is in line with several literature studies [19,23,25,30]. Other works [18,20,21] have reported a higher prevalence of epilepsy, which could depend on a selection bias toward patients with already established neurological problems.

The distribution of patients in the epileptic cohort slightly favored the male sex (M vs. F: 6% vs. 3.6%, *p*-value 0.16), although without statistical significance, as observed by other authors [20,23,25].

**Figure 1:**
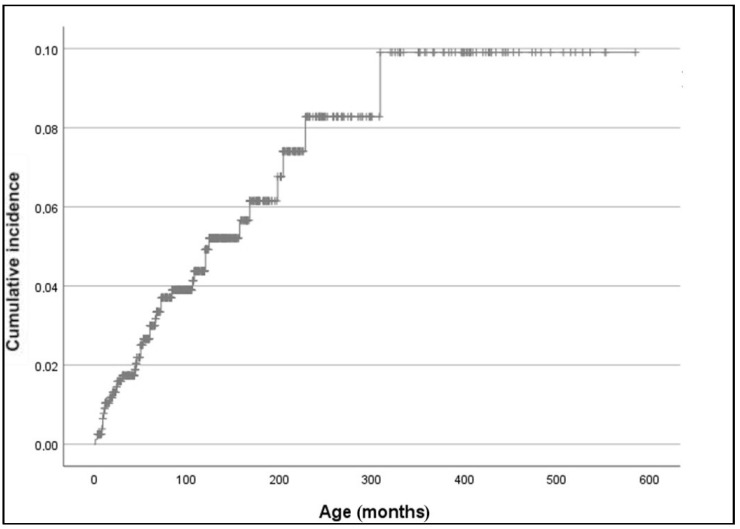
Total cumulative incidence of epilepsy in NF1 patients (Kaplan–Meier estimate).

Considering the heterogeneity of our cohort in terms of follow-up duration, we also calculated the cumulative incidence of epilepsy using Kaplan–Meier analysis: the cumulative incidence of epilepsy in our population was 4% at 100 months of age (8.3 years) and 9.9% at 300 months (20.5 years) (standard error 7.879%) (Figure 1). However, the latter finding should probably be considered an overestimation, as an early loss to follow-up should be more likely to happen in subjects who do not develop complications.

Family history data on NF1 were available for 36 epileptic patients, of whom 9 had a familial NF1 (25%) (maternal in 5 out of 9, paternal in 4 out of 9), and for 267 out of 706 patients (37.9%) in the nonepileptic cohort (maternal in 58% of cases, paternal in 42%). This difference between the two cohorts was not statistically significant, in agreement with other recent studies [23,25].

No parent-of-origin effect was observed in the 9 epileptic cases with familial NF1, unlike what has been previously reported by Ostendorf and colleagues, who found that the nonsporadic NF1 individuals with epilepsy had more frequently inherited the NF1 from the mother.

Epilepsy family history (first- and second-degree relatives) was ascertained in 3 out of 36 (8.3%) epileptic patients, being significantly higher than the 1.98% (14 out of 784) of the nonepileptic cohort (*p*-value 0.0389), in agreement with what has been previously reported [20,23]. However, in our cohort, two out of the three patients with a family history of epilepsy presented with cerebral structural abnormalities, and, in one patient, a family history for NF1 was documented as well (Table S1). Therefore, we did not observe a positive correlation with family history of epilepsy in epileptic NF1 patients with nonstructural seizures, i.e., with negative neuroimaging regardless of a family history for NF1.

### 2.2. Epileptic Phenotype

The neurological phenotype of NF1 patients is described in Table 2 and Table S1.

The mean age of seizure onset was 8 years and 8 months, with a minimum of 1 month and a maximum of 25 years and 7 months (Table S1). Previous retrospective observational studies have reported different ages of onset (5 years and 5 months [23], 6 years [25], 6 years and 6 months [21], and 9.5 years [20], respectively), probably reflecting the differences in ascertainment and inclusion criteria. In 21 patients, seizures began before the first visit to our NF1 clinic, while 16 of them developed seizures later during the follow-up.

Among the 32 patients whose neurophysiopathological data were available, the electro-clinical diagnosis was focal epilepsy in 13 cases (40%), generalized epilepsy in 17 cases (53.1%), and epileptic encephalopathy (West’s syndrome) in the remaining 2 (6.2%) (Table 2). As such, it appears that there was no prevalence of focal epilepsies compared to generalized ones. Although some previous works have shown that most seizures in NF1 patients are often classified as focal to bilateral tonic-clonic seizures [17,18,23,31], only 3 of our patients presented in this way. Our data suggest that there is not a specific seizure pattern in these patients, as reported by Korf et al. [17], and later confirmed by other studies [20,21,25]. The examination of the interictal EEGs did not show any specific, recurrent electrical activity patterns either (Table S1). However, it should be noted that these neurophysiological records were gathered over a large time interval, characterized by a constant evolution of classification criteria and diagnostic capabilities, thus introducing a possible bias into the interpretation of these electro-clinical data at the time of the diagnosis.

Out of 33 patients whose treatment information was available, only 3 (8.5%) underwent surgical treatment for epilepsy; all these individuals also underwent drug treatment, for two of them in monotherapy, while for the other in triple therapy (Table S1). Most of the epileptic patients were treated with at least one anti-seizure medication (78.8%, 26 out of 33); only 3 of 26 (11.5%) needed more than one drug to obtain seizure freedom. At the end of the follow up, 30 patients (85.7%) had been seizure-free for at least one year, while a minority (5 out of 35) experienced relapsing seizures not controlled by antiseizure medications.

Both patients diagnosed with West’s syndrome, whose association with NF1 had already been established [32], showed a favorable outcome, with no seizures for at least one year after diagnosis and treatment, unlike what was previously observed in patients with West’s syndrome from other cohorts, where seizures could not be controlled by polytherapy [18,20,21].

### 2.3. Neurological Comorbidities

The results of the neurological data and neuroimaging investigations performed in the two cohorts are described in Table 3.

Concerning the neurocognitive phenotype of the cohort with epilepsy, we found a higher incidence of intellectual disability and/or developmental delay, as well as of isolated learning disabilities, as already reported by other studies [18,23,25]. In fact, up to 37.8% (14 out of 37) of patients with epilepsy were affected by developmental delay and/or different degrees of cognitive impairment, compared with the 15.4% (112 out of 712) of nonepileptic patients; such a difference was statistically significant (*p*-value of 0.0009). In addition, even when not directly associated with psychomotor delay/cognitive deficit and/or language delay, learning disabilities were more frequent in the epileptic cohort (29.7%, 11 out of 37) than in the NF1 patients without epilepsy (15.1%, 113 out of 747), with a statistically significant difference between the two cohorts (*p*-value 0.027).

Thirty-four of the 37 (91.9%) patients with epilepsy underwent at least one neuroimaging examination during their lifetime (2 CT, 32 brain MRI, Table 2). Among them, 29.4% (10 out of 34) had a negative brain imaging (except for the presence of UBOs), while 70.6% (24 out of 34) displayed one or more brain abnormalities (neuroimaging was considered altered in the presence of any anomaly such as OPG, non-OPG brain neoplasia, hydrocephalus, cortical dysplasia, and vascular disease). In the nonepileptic NF1 cohort, only 378 out of 747 patients underwent brain imaging (MRI or CT). Among the tested subjects, those with a negative result (except for UBOs) were 52.9% (200 out of 378), while 178 out of 378 (47.1%) had a pathological finding. In this regard, it should be noted that, considering that NF1 patients referred to our Center perform brain imaging only in the presence of neurological symptoms, the proportion of pathological neuroimaging in the nonepileptic group could be overestimated.

The presence of a brain anomaly at neuroimaging was therefore observed more frequently in the epileptic cohort (*p*-value of 0.0038) compared with the nonepileptic one, in agreement with previously published data [21,23]. It should also be pointed out that 3 out of 24 patients with structural epilepsy showed isolated brain anomalies that are not typically related to NF1 (Table 2). Thus, it can be estimated that approximately 62% of the cases of seizure in NF1 patients of our cohort arose in the context of NF1-related neuroradiological findings, which primarily include hydrocephalus, cerebral vasculopathies, and brain neoplasms.

Remarkably, the prevalence of hydrocephalus (14.7%), cerebral vasculopathies (14.7%), and non-OPG brain neoplasm (29.4%) in the patients with epilepsy was found significantly higher than that of the non-epileptic cohort (5.3%, 1.85%, and 12.9%, respectively). In particular, binary logistic regression (Table 4) showed that the main predictors of epilepsy among neurological comorbidities are indeed encephalic vasculopathies (RR 6.17; 95% CI: 1.67–22.78) and hydrocephalus (RR 3.293; 95% CI: 1.04–10.4).

Cerebrovascular anomalies have been reported in the literature in about 4% of patients with NF1 [33], and a recent study [25] found no significant differences between patients with or without seizures. In our work, instead, they were remarkably more frequent in the epileptic group (14.7%) compared with the nonepileptic cohort (1.85%), resulting to be the strongest predictive variable in multivariate analysis (Table 4).

The incidence of hydrocephalus in our NF1 cohort appears to be slightly greater than what has been previously reported (1.4% according to Hirabaru et al. [33]), being found in 5.3% of the nonepileptic cohort and even as high as 16.2% in patients with seizures.

Non-OPG brain neoplasms were frequent in both cohorts as well, showing in 27% of epileptic patients and 12.9% of the nonepileptic patients who underwent brain imaging, which is higher than the 3.4% reported by Hirabaru.

The prevalence of optic pathway glioma (OPG) proved to be approximatively comparable (32.4% versus 34.9%) amongst the two cohorts, confirming the absence of correlation between the two clinical manifestations, as previously reported [24].

Other central nervous system anomalies showed altogether similar frequencies in both groups (*p*-value 0.17). In particular, we did not confirm the incidence of cortical malformations reported in epileptic patients with NF1 by Vivarelli et al. [18]. As these authors pointed out themselves, their estimate could have suffered from a relatively small sample and a selection bias due to the referral criteria of their clinic.

In about 29.4% of epileptic patients (10 out of 34), neuroimaging was normal; therefore, considering only patients with “nonstructural” epilepsy, its prevalence in the global cohort resulted to be 1.27% (10/784, CI: 0.49–2.06%), which is compatible with the overall occurrence in the general population. This finding, despite showing a relative increase in the proportion of nonstructural epilepsy in NF1 patients, does not clarify the hypothesis, already proposed by other authors [20,22,25], that specific mechanisms, inherent to NF1 and neurofibromin cellular function, may determine an increased risk of epilepsy besides the higher frequency of structural cerebral abnormalities.

Among the patients who performed MRI, UBOs were detected in 24 out of the 32 epileptic patients (75%) and 193 out of 334 of the nonepileptic group (57.8%). Such incidences are compatible with previous reports [23,34]. The lower prevalence in the nonepileptic group could be explained by the fact that images of epileptic patients have been reviewed by NF1-specialized neuroradiologists, who are particularly experts in recognizing these signal alterations.

The complete neuroradiological phenotype of epileptic patients is described in Table S1.

Regarding the response to drug treatment, 30 out of 37 patients have been seizure-free for at least one year: of these individuals, 7 did not require any drugs, while the other 23 used only one anti-seizure medication. Despite more than half of our patients having at least one neuroimaging anomaly, the seizure outcome was favorable overall, in contrast to polytherapy and drug resistance previously reported in the presence of brain anomalies [20]. Drug-resistant epilepsy, defined as a failure to respond to more than two anti-seizure medications, occurred in 2 out of our 37 patients; three other patients did not respond to only one trial of treatment. Such a proportion of uncontrolled seizures is compatible with what has been previously described [35]. An abnormality in neuroimaging was observed in both the drug-resistant patients (i.e., hydrocephalus in one patient and a non-OPG brain neoplasia in another case), but the sample was too small to infer any correlation between structural abnormalities and drug resistance. Neurosurgery was performed on three of our patients, one of which with partial resection of the temporal lobe with subsequent seizure freedom, as reported in previous studies [36], while the other two underwent resection of encephalic neoplasia (one with seizure-free outcome, the other without benefit on seizure control).

### 2.4. Molecular Diagnosis

The causative molecular defects identified in the *NF1* gene in our patients are reported in Table 5.

In six of the patients from the epileptic cohort (10.8%) and 214 of patients without seizures, no analysis of the *NF1* gene could be performed. Among the tested patients, in 6 (18.2%) and 61 (12.2%), respectively, only the 17q11 microdeletion could be excluded.

Microdeletion 17q11 was found in two (8.3%) of the tested epileptic patients and 22 (4.9%) from the nonepileptic cohort; this difference was not statistically significant (*p*-value 0.3533). In the literature, there are discordant data on the correlation between epilepsy and the microdeletion including the *NF1* gene. This mutation, in fact, is absent in all epileptic patients studied by Van Minkelen et al. [37], while Santoro and colleagues [23] detected it in 18% of patients with seizures. Some authors suggested that the pathogenesis of seizures in patients with the 17q11 microdeletion could be associated with the other genes involved in this rearrangement [23,25].

With the exception of 17q11 microdeletion, no other mutations associated with a specific phenotypic correlation (such as those affecting codons 844–848, 992, 1038, and 1809 [7,8,9,10]) were found in our cohort of epileptic patients.

The majority of both epileptic (70.8%) and nonepileptic (64.5%) NF1 patients with a molecular diagnosis exhibited either a loss-of-function mutation or the 17q11 microdeletion. Notably, only 4.2% of epileptic patients carried a missense mutation [38], a prevalence way lower than the 14.4% of the nonepileptic cohort, which is adherent to the literature data [39]. Inversely, a relative increase in splice site mutations was observed in the epileptic cohort (25% vs. 15.6%). However, further studies with larger sample sizes are needed to clarify whether such a shift in the prevalence of missense and splice site mutations in the epileptic cohort could correlate with the neurological phenotype.

## 3. Materials and Methods

### 3.1. Patients

We retrospectively evaluated the clinical data from a cohort of 2074 unselected patients, referred to the NF1 Clinic of the University of Padova between October 1979 and December 2020, with a clinical diagnosis or suspicion of NF1. The inclusion criteria consisted of the clinical and/or molecular diagnosis of NF1 during the first evaluation or later during the follow-up (according to the established International Criteria [12,13]), while mosaic or segmental NF1 (defined as the presence of diagnostic criteria limited to one body area without crossing the midline [40]) patients were excluded. In order to avoid possible referral biases, we included only patients whose first evaluation was performed before the age of 13 years old.

In the 784 patients matching the inclusion criteria, we collected general clinical data, including age, sex, family history, NF1 genotype, brain-imaging reports, and history of epilepsy and of other comorbidities. We particularly focused on the neurological phenotype, recording the prevalence of UBOs, headache, hydrocephalus, vasculopathies (such as moyamoya and vessel ectasia, aneurysm, hypoplasia, and narrowing), OPG, brain tumors, and other brain abnormalities (such as cortical dysplasia or hippocampal dysplasia). Regarding the intellectual phenotype, we defined intellectual disability (ID) as an IQ lower than 70 and learning disabilities as significant difficulties in the acquisition and use of listening skills, oral expression, reading, reasoning, and/or mathematics; we have also reported behavior abnormalities (e.g., ADHD).

In regard to epilepsy, in compliance with the definition of the International League Against Epilepsy (ILAE) [41,42,43], patients were considered epileptic if they had exhibited at least either two unprovoked seizures > 24 h apart from each other, or a single unprovoked seizure associated with a high likelihood of a persistently lowered seizure threshold and, therefore, a high recurrence risk, or an epilepsy syndrome. Patients with febrile seizures only were excluded from the epileptic cohort. Drug-resistant epilepsy was defined as the “failure of adequate trials of two tolerated, appropriately chosen, and used antiepileptic drug schedules (whether as monotherapies or in combination) to achieve sustained seizure freedom,” according to the ILAE definition.

We then collected family history, age of seizure onset, semiology, electroencephalography (EEG) reports, anti-seizure medications, and seizure outcomes (seizure-free for at least one year, presence of seizures in the last year or unknown).

### 3.2. Follow-Up Protocol

NF1 patients undergo a follow-up program consisting of annual clinical examinations, annual specialist ophthalmologic visits, and blood pressure measurements. Neuroimaging and other specific investigations (such as abdominal ultrasonography, echocardiography, and EEG) were proposed in the presence of a proper clinical indication [44,45].

### 3.3. Molecular Analysis

Molecular analysis of the *NF1* gene in the patients was performed as previously described [46]; particularly, over the years, different methods have been employed, including: mutation screening approaches (HRM analysis) [47], cDNA analysis, and a NGS-based sequencing protocol analyzing NF1, SPRED1, and other genes associated with CALMS. Screening for whole gene deletions was carried out through FISH analysis, as previously reported [48], or multiplex ligation-dependent probe amplification (MLPA) analysis, which also allowed the detection of single/multiexon intragenic deletions/duplications (SALSA MLPA kits P081/P082 MRC Holland, Amsterdam, The Netherlands).

When DNA was available from other family members, segregation analysis was carried out through bidirectional Sanger sequencing or MLPA analysis depending on the variation.

### 3.4. Statistical Analyses

Dichotomic variables were compared using Fisher’s exact test. Quantitative variables were compared with Student’s *t*-test. The Kaplan–Meier survival curve was used to estimate the cumulative incidence of epilepsy, accounting for interval censoring [49]. Multiple logistic regression was used to evaluate whether the incidence of epilepsy was significantly different according to sex, the presence of neurological abnormalities, a positive family history for NF1, or epilepsy. All *p* values were calculated at a 95% CI. Statistical analyses were performed using IBM SPSS Statistics for Windows, Version 27.0.

## 4. Conclusions

We confirmed the epidemiological data reported in the most recent literature on the incidence of epilepsy in NF1; in fact, a total prevalence of 4.72% was observed in our unselected cohort, with no statistically significant differences between sexes. A specific pattern of seizures in these patients was not identified, in agreement with what has already been described by other studies. In line with other works, we also found a higher incidence of intellectual disability and/or developmental delay in patients with NF1 and epilepsy, as well as of learning disabilities.

Our observational study confirms that epilepsy is a neurological complication of NF1, mainly associated with intracranial structural complications (70.6% of cases). The principal predisposing factors for the development of seizures in our cohort were the presence of cerebral vasculopathies and hydrocephalus.

In addition, we found that only 1.27% of NF1 patients developed seizures in the absence of structural brain abnormalities, arguing against the hypothesis that specific mechanisms, inherent to NF1 and neurofibromin cellular function, may determine an increased risk of epilepsy in this condition. This finding may have relevant consequences on the clinical management of patients.

Regarding possible genotype–phenotype correlations, we did not document any statistically significant difference in the mutational spectrum between the cohort of epileptic and nonepileptic patients, not even for microdeletion 17q11. However, an interesting shift in the prevalence of missense and splice site mutations, favoring the latter, was found in epileptic patients compared with both our nonepileptic cohort and the general literature data. Further studies with a larger number of genotyped patients are needed to confirm this finding and evaluate any further genotype–phenotype correlations.

## Figures and Tables

**Table 1:** Epidemiologic features of NF1 patients with and without epilepsy.

	Total Cohort (784)	Patients without Epilepsy (747)	Patients with Epilepsy (37)	*p*-Value
Sex (Females/Males)	371/413	358/389	13/24	0.18
Mean age at first visit (months)	58.25	57.59	69.57	0.10 < *p* < 0.20
	SD ^1^: 43.7	SD: 43.4	SD: 49.4	
Follow-up mean length (months)	82	81	105	0.10 < *p* < 0.20
	SD: 101	SD: 101	SD: 96	
NF1 family history	276 out of 742 (37.2%)	267 out of 706 (37.8%)	9 out of 36 (25%)	0.1568
Mother/Father	116/160	112/153	5/4	
Epilepsy family history	17 out of 742 (2.29%)	14 out of 706 (1.98%)	3 out of 36 (8.3%)	0.0389

^1^ SD: Standard Deviation.

**Table 2:** NF1 patients with epilepsy ^1^.

ID-Sex	NF1 Mutation (NM_000267.3)	Neuro-Cognitive Phenotype	Brain MRI Imaging ^§,^°	Type of Seizures
1-F	Not tested	LD	NA	Unknown
2-M	c.3266delA p.(Gly1090fs*1095) Null	LD	OPG; UBOs	Focal (MO, IA)
3-M	Not tested	LD+ADHD	Normal	Focal
4-F	Not tested	Normal	UBOs	Generalized
5-M	c.5471T > G p.(Ile1824Ser) Missense	mild ID, LD	Brainstem (pons)/left cerebellar peduncle nodular lesion; Hydrocephalus; UBOs	Combined generalized and focal
6-M	c.2273_2274insT p.(Arg758Serfs*10) Null	DD, mild ID, LD	Low-grade astrocytoma (left frontal lobe)	Focal
7-M	Not tested	DD	Hydrocephalus	Generalized
8-M	Not tested	DD	Cerebral angioma (occipital lobe) ^§^	Generalized (MO, TC)
9-F	c.662G > A p.(Trp221*) Null	LD, ID, AD	Stroke; OPG; moyamoya; UBOs	Focal (MO)
10-M	c.369delC p.(Cys124Valfs*41) Null	ID	Suprasellar lesion; OPG; UBOs	Focal
11-M	c.3113 + 1G > A r.2991_3113del p.(Tyr998_Arg1038del) Splice site	DD, mild ID	OPG; UBOs	Generalized, (MO, myotonic-atonic)
12-M	Not tested	Normal	NA	Generalized (MO, TC)
13-F	c.5839 C > T p.(Arg1947*) Null	LD	Hydrocephalus; OPG; Absent MCA; Hypoplasic ICA	Generalized
14-M	c.541C > T p.(Gln181*) Null	LD	UBOs	Generalized
15-M	Not tested	NA (lost to FU at 6 months)	Left basal ganglia glioma; Hydrocephalus; OPG; UBOs	Unknown
16-M	c.889-2A > G r.? p.? Splice site	DD, LD	UBOs	Focal (MO)
17-M	c.532G > T p.(Glu178*) Null	DD	NA	Generalized (absence)
18-F	c.6220_ 6221insCAACAATTCCCTT p.(Asp2074Alafs*29) Null	DD, mild ID, LD	Left centrum semiovalis signal alterations, compatible with perinatal brain injury °; UBOs	Spasms
19-M	c.3233_3234insT p.(Leu1079Thrfs*10) Null	Normal	Low-grade cerebellar astrocytoma; left carotid-jugular arteriovenous fistula; UBOs	Focal
20-M	c.204+1G > T r.100_204del p.(Val34_Met68del) Splice site	LD+ ADHD, BD, A	Brainstem (pons) capillary telangiectasia; UBOs	Generalized (absence)
21-F	c.185delT p.(Leu62*) Null	DD, LD	UBOs	Generalized
22-M	Not tested	mild ID	Olfactory cortex lesion; UBOs	Focal (MO)
23-F	17q11 microdeletion	LD	Brainstem low-grade lesion; UBOs	Focal
24-F	c.4269+1G > A Splice site	Normal	Right thalamic lesion; periventricular nodular heterotopia; UBOs	Combined generalized and focal
25-M	17q11 microdeletion	mild ID	Hydrocephalus; Arnold Chiari I malformation; OPG; UBOs	Generalized
26-F	c.1185+1 G > A r.1063_1185del p.(Asn355_Lys395del) Splice site	LD	Normal	Generalized (MO, TC)
27-M	c.1945G > T p.(Glu649*) Null	mild ID	Incomplete hippocampal inversion °; OPG; UBOs	Combined generalized and focal
28-M	c.82C > T p.(Gln28*) Null	Normal	UBOs	Focal (MO, clonic)
29-M	Not tested	Normal	Cerebral cortical calcification ° (left parietal lobe) ^§^	Unknown
30-F	Negative (NGS+MLPA)	LD	Normal	Generalized (absence)
31-M	Not tested	Normal	Subcortical signal enhancement; Lateral ventricles asymmetry; UBOs	Generalized (absence)
32-M	c.3457_3460del p.(Leu1153Metfs*4) Null	Normal	OPG; lentiform nucleus low-grade lesion; UBOs	Focal (behavior arrest)
33-M	c.2991-1G > A r.? p.? Splice site	ID	OPG; UBOs	Generalized (absence)
34-F	c.7352delC p.(Pro2451Leufs*17) Null	DD	OPG; UBOs	Spasms
35-M	Not tested	Normal	UBOs	Generalized
36-F	Not tested	Normal	Left trigone hypodense lesion	Generalized (myoclonic absence)
37-F	c.291delA p.(Gln97Hisfs*6) Null	Normal	UBOs	Generalized, (MO, TC)

^1^ LD: Learning disabilities; DD: developmental delay; ID: intellectual disability; ADHD: attention deficit hyperactivity disorder; BD: bipolar disorder; AD: anxiety disorder; OPG: optic pathways glioma; UBO: unidentified bright object; NA: not available; RH: recurrent headaches; MO: motor onset; IA: impaired awareness; TC: tonic-clonic; ^§^ Brain imaging was performed through TC scan instead of MRI; ° structural NF1-unrelated anomaly.

**Table 3:** Neurologic phenotype.

	Nonepileptic Patients	Epileptic Patients	*p*-Value *
Developmental delay and/or intellectual disability	112 out of 747 (15%)	14 out of 37 (37.8%)	0.0009 *
Learning disabilities with normal intellect	113 out of 747 (15.1%)	11 out of 37 (29.7%)	0.0337 *
Recurrent headache	101 out of 747 (13.5%)	13 out of 37 (35.1%)	0.0011 *
Pathologic imaging (except for UBOs)	178 out of 378 (47.1%)	24 out of 34 (70.6%)	0.0114 *
Hydrocephalus	20 out of 378 (5.3%)	5 out of 34 (14.7%)	0.045 *
Cerebral vasculopathies	7 out of 378 (1.85%)	5 out of 34 (14.7%)	0.0015 *
OPG	132 out of 378 (34.9%)	11 out of 34 (32.4%)	0.8521
Brain neoplasia other than OPG (%)	49 out of 378 (12.9%)	10 out of 34 (29.4%)	0.0177 *
Other CNS anomalies	34 out of 378 (9%)	6 out of 34 (17.6%)	0.124
UBOs	193 out of 334 (57.8%)	24 out of 32 (75%)	0.062

* *p*-value < 0.05.

**Table 4:** Binary logistic regression (multivariate analysis).

	*p*-Value	OR	95% C.I. OR	
			Lower	Upper
Sex	0.294	1.514	0.697	3.288
NF1 family history	0.970	0.985	0.432	2.241
Epilepsy family history	0.970	1.043	0.113	9.632
Hydrocephalus	0.042	3.293	1.043	10.403
Cerebral vasculopathies	0.006	6.173	1.673	22.781
OPG	0.755	0.878	0.389	1.983
Non OPG Neoplasia	0.413	1.503	0.567	3.983

**Table 5:** Genotype.

	Nonepileptic NF1 Patients (*n* = 747)	Epileptic NF1 Patients (*n* = 37)	
Untested	214	4	
(% of cohort)	(28.6)	(10.8)	
At least one test	525	31	
(% of cohort)	(70.2)	(83.8)	
Tested for 17q11 microdeletion only	61	6	
(% of cohort)	(8.2)	(18.2)	
			***p*-value**
17q11 microdeletion	22	2	
(% of cohort)	(2.9)	(5.4)	0.3145
(% of confirmed molecular defects)	(4.9)	(8.3)	0.3533
Identified mutation	421	22	
(% of cohort)	(56.3)	(59.4)	
(% of MLPA and NGS analyses)	(90.7)	(88)	
Nonsense or frameshift mutation	264	15	
(% of cohort)	(35.3)	(40.5)	0.5981
(% of confirmed molecular defects)	(59.6)	(62.5)	0.8339
Missense mutation	64	1	
(% of cohort)	(8.6)	(2.7)	0.3545
(% of confirmed molecular defects)	(14.4)	(4.2)	0.2276
Splicing mutation	69	6	
(% of cohort)	(9.2)	(16.2)	0.6128
(% of confirmed molecular defects)	(15.6)	(25)	0.2494
In frame mutation	8		
(% of cohort)	(1.1)	-	1
(% of confirmed molecular defects)	(1.8)		
Exonic/multiexonic deletion	12		
(% of cohort)	(1.6)	-	1
(% of confirmed molecular defects)	(2.7)		
Other	4		
(% of cohort)	(0.5)	-	1
(% of confirmed molecular defects)	(0.9)		
No causative mutation or CNV detected	43	3	
(% of tested patients)	(8.2)	(9.1)	

## Data Availability

The data presented in this study are available on request from the corresponding author.

## Supplementary Materials

The following are available online at https://www.mdpi.com/article/10.3390/cancers13246336/s1, Table S1: Phenotypic and genotypic characterization of epileptic NF1 patients.

## References

[1] Carey J.C., Baty B.J., Johnson J.P., Morrison T., Skolnick M., Kivlin J. (1986). The Genetic Aspects of Neurofibromatosis. Ann. N. Y. Acad. Sci..

[2] Rasmussen S.A., Friedman J.M. (2000). NF1 Gene and Neurofibromatosis 1. Am. J. Epidemiol..

[3] Evans D.G., Howard E., Giblin C., Clancy T., Spencer H., Huson S.M., Lalloo F. (2010). Birth Incidence and Prevalence of Tumor-Prone Syndromes: Estimates from a UK Family Genetic Register Service. Am. J. Med. Genet. A.

[4] Kallionpää R.A., Uusitalo E., Leppävirta J., Pöyhönen M., Peltonen S., Peltonen J. (2018). Prevalence of Neurofibromatosis Type 1 in the Finnish Population. Genet. Med..

[5] Gutmann D.H., Ferner R.E., Listernick R.H., Korf B.R., Wolters P.L., Johnson K.J. (2017). Neurofibromatosis Type 1. Nat. Rev. Dis. Primer.

[6] Wang W., Wei C.-J., Cui X.-W., Li Y.-H., Gu Y.-H., Gu B., Li Q.-F., Wang Z.-C. (2021). Impacts of NF1 Gene Mutations and Genetic Modifiers in Neurofibromatosis Type 1. Front. Neurol..

[7] Koczkowska M., Callens T., Gomes A., Sharp A., Chen Y., Hicks A.D., Aylsworth A.S., Azizi A.A., Basel D.G., Bellus G. (2019). Expanding the Clinical Phenotype of Individuals with a 3-Bp in-Frame Deletion of the NF1 Gene (c.2970_2972del): An Update of Genotype–Phenotype Correlation. Genet. Med..

[8] Pinna V., Lanari V., Daniele P., Consoli F., Agolini E., Margiotti K., Bottillo I., Torrente I., Bruselles A., Fusilli C. (2015). P. Arg1809Cys Substitution in Neurofibromin Is Associated with a Distinctive NF1 Phenotype without Neurofibromas. Eur. J. Hum. Genet..

[9] Trevisson E., Morbidoni V., Forzan M., Daolio C., Fumini V., Parrozzani R., Cassina M., Midena E., Salviati L., Clementi M. (2019). The Arg1038Gly Missense Variant in the *NF1* Gene Causes a Mild Phenotype without Neurofibromas. Mol. Genet. Genom. Med..

[10] Koczkowska M., Chen Y., Callens T., Gomes A., Sharp A., Johnson S., Hsiao M.-C., Chen Z., Balasubramanian M., Barnett C.P. (2018). Genotype-Phenotype Correlation in NF1: Evidence for a More Severe Phenotype Associated with Missense Mutations Affecting NF1 Codons 844–848. Am. J. Hum. Genet..

[11] Pasmant E., Sabbagh A., Spurlock G., Laurendeau I., Grillo E., Hamel M.-J., Martin L., Barbarot S., Leheup B., Rodriguez D. (2010). NF1 Microdeletions in Neurofibromatosis Type 1: From Genotype to Phenotype. Hum. Mutat..

[12] (1988). National Institutes of Health Consensus Development Conference Statement: Neurofibromatosis. Bethesda, MD, USA, July 13–15, 1987. Neurofibromatosis.

[13] Legius E., Messiaen L., Wolkenstein P., Pancza P., Avery R.A., Berman Y., Blakeley J., Babovic-Vuksanovic D., Cunha K.S., Ferner R. (2021). Revised Diagnostic Criteria for Neurofibromatosis Type 1 and Legius Syndrome: An International Consensus Recommendation. Genet. Med..

[14] Bayat M., Bayat A. (2020). Neurological Manifestations of Neurofibromatosis: A Review. Neurol. Sci..

[15] Vogel A.C., Gutmann D.H., Morris S.M. (2017). Neurodevelopmental Disorders in Children with Neurofibromatosis Type 1. Dev. Med. Child Neurol..

[16] Nix J.S., Blakeley J., Rodriguez F.J. (2020). An Update on the Central Nervous System Manifestations of Neurofibromatosis Type 1. Acta Neuropathol..

[17] Korf B.R., Carrazana E., Holmes G.L. (1993). Patterns of Seizures Observed in Association with Neurofibromatosis 1. Epilepsia.

[18] Vivarelli R., Grosso S., Calabrese F., Farnetani M., Di Bartolo R., Morgese G., Balestri P. (2003). Epilepsy in Neurofibromatosis 1. J. Child Neurol..

[19] Hsieh H.-Y., Fung H.-C., Wang C.-J., Chin S.-C., Wu T. (2011). Epileptic Seizures in Neurofibromatosis Type 1 Are Related to Intracranial Tumors but Not to Neurofibromatosis Bright Objects. Seizure.

[20] Ostendorf A.P., Gutmann D.H., Weisenberg J.L.Z. (2013). Epilepsy in Individuals with Neurofibromatosis Type 1. Epilepsia.

[21] Pecoraro A., Arehart E., Gallentine W., Radtke R., Smith E., Pizoli C., Kansagra S., Abdelnour E., McLendon R., Mikati M.A. (2017). Epilepsy in Neurofibromatosis Type 1. Epilepsy Behav..

[22] Stafstrom C.E., Staedtke V., Comi A.M. (2017). Epilepsy Mechanisms in Neurocutaneous Disorders: Tuberous Sclerosis Complex, Neurofibromatosis Type 1, and Sturge–Weber Syndrome. Front. Neurol..

[23] Santoro C., Bernardo P., Coppola A., Pugliese U., Cirillo M., Giugliano T., Piluso G., Cinalli G., Striano S., Bravaccio C. (2018). Seizures in Children with Neurofibromatosis Type 1: Is Neurofibromatosis Type 1 Enough?. Ital. J. Pediatr..

[24] Bernardo P., Cinalli G., Santoro C. (2020). Epilepsy in NF1: A Systematic Review of the Literature. Childs Nerv. Syst..

[25] Serdaroglu E., Konuskan B., Karli Oguz K., Gurler G., Yalnizoglu D., Anlar B. (2019). Epilepsy in Neurofibromatosis Type 1: Diffuse Cerebral Dysfunction?. Epilepsy Behav..

[26] Cui Y., Costa R.M., Murphy G.G., Elgersma Y., Zhu Y., Gutmann D.H., Parada L.F., Mody I., Silva A.J. (2008). Neurofibromin Regulation of ERK Signaling Modulates GABA Release and Learning. Cell.

[27] Ren M., Li K., Wang D., Guo J., Li J., Yang G., Long X., Shen W., Hu R., Wang X. (2016). Neurofibromin Regulates Seizure Attacks in the Rat Pilocarpine-Induced Model of Epilepsy. Mol. Neurobiol..

[28] Rizwan G., Sabetghadam A., Wu C., Liu J., Zhang L., Reid A.Y. (2019). Increased Seizure Susceptibility in a Mouse Model of Neurofibromatosis Type 1. Epilepsy Res..

[29] Sabetghadam A., Wu C., Liu J., Zhang L., Reid A.Y. (2020). Increased Epileptogenicity in a Mouse Model of Neurofibromatosis Type 1. Exp. Neurol..

[30] Madubata C.C., Olsen M.A., Stwalley D.L., Gutmann D.H., Johnson K.J. (2015). Neurofibromatosis Type 1 and Chronic Neurological Conditions in the United States: An Administrative Claims Analysis. Genet. Med..

[31] Caraballo R., Portuondo E., Fortini P. (2015). Neurofibromatosis and Epilepsy. J. Pediatr. Epilepsy.

[32] Motte J., Billard C., Fejerman N., Sfaello Z., Arroyo H., Dulac O. (1993). Neurofibromatosis Type One and West Syndrome: A Relatively Benign Association. Epilepsia.

[33] Hirabaru K., Matsuo M. (2018). Neurological Comorbidity in Children with Neurofibromatosis Type 1. Pediatr. Int..

[34] Sabol Z., Resić B., Gjergja Juraski R., Sabol F., Kovac Sizgorić M., Orsolić K., Ozretić D., Sepić-Grahovac D. (2011). Clinical Sensitivity and Specificity of Multiple T2-Hyperintensities on Brain Magnetic Resonance Imaging in Diagnosis of Neurofibromatosis Type 1 in Children: Diagnostic Accuracy Study. Croat. Med. J..

[35] Gales J., Prayson R.A. (2017). Hippocampal Sclerosis and Associated Focal Cortical Dysplasia-Related Epilepsy in Neurofibromatosis Type, I. J. Clin. Neurosci..

[36] Barba C., Jacques T., Kahane P., Polster T., Isnard J., Leijten F.S.S., Ozkara C., Tassi L., Giordano F., Castagna M. (2013). Epilepsy Surgery in Neurofibromatosis Type 1. Epilepsy Res..

[37] Van Minkelen R., van Bever Y., Kromosoeto J.N.R., Withagen-Hermans C.J., Nieuwlaat A., Halley D.J.J., van den Ouweland A.M.W. (2014). A Clinical and Genetic Overview of 18 Years Neurofibromatosis Type 1 Molecular Diagnostics in the Netherlands. Clin. Genet..

[38] Brinckmann A., Mischung C., Bässmann I., Kühnisch J., Schuelke M., Tinschert S., Nürnberg P. (2007). Detection of NovelNF1 Mutations and Rapid Mutation Prescreening with Pyrosequencing. Electrophoresis.

[39] Messiaen L.M., Wimmer K., Kaufmann D. (2008). NF1 Mutational Spectrum. Monographs in Human Genetics.

[40] Listernick R., Mancini A.J., Charrow J. (2003). Segmental Neurofibromatosis in Childhood. Am. J. Med. Genet..

[41] Fisher R.S., Cross J.H., D’Souza C., French J.A., Haut S.R., Higurashi N., Hirsch E., Jansen F.E., Lagae L., Moshé S.L. (2017). Instruction Manual for the ILAE 2017 Operational Classification of Seizure Types. Epilepsia.

[42] Fisher R.S., Acevedo C., Arzimanoglou A., Bogacz A., Cross J.H., Elger C.E., Engel J., Forsgren L., French J.A., Glynn M. (2014). ILAE Official Report: A Practical Clinical Definition of Epilepsy. Epilepsia.

[43] Scheffer I.E., Berkovic S., Capovilla G., Connolly M.B., French J., Guilhoto L., Hirsch E., Jain S., Mathern G.W., Moshé S.L. (2017). ILAE Classification of the Epilepsies: Position Paper of the ILAE Commission for Classification and Terminology. Epilepsia.

[44] Ferner R.E., Huson S.M., Thomas N., Moss C., Willshaw H., Evans D.G., Upadhyaya M., Towers R., Gleeson M., Steiger C. (2007). Guidelines for the Diagnosis and Management of Individuals with Neurofibromatosis 1. J. Med. Genet..

[45] Trevisson E., Cassina M., Opocher E., Vicenzi V., Lucchetta M., Parrozzani R., Miglionico G., Mardari R., Viscardi E., Midena E. (2017). Natural History of Optic Pathway Gliomas in a Cohort of Unselected Patients Affected by Neurofibromatosis 1. J. Neurooncol..

[46] Morbidoni V., Baschiera E., Forzan M., Fumini V., Ali D.S., Giorgi G., Buson L., Desbats M.A., Cassina M., Clementi M. (2021). Hybrid Minigene Assay: An Efficient Tool to Characterize MRNA Splicing Profiles of NF1 Variants. Cancers.

[47] Forzan M., Salviati L., Pertegato V., Casarin A., Bruson A., Trevisson E., Di Gianantonio E., Clementi M. (2010). Is CFTR 621+3 A > G a Cystic Fibrosis Causing Mutation?. J. Hum. Genet..

[48] Riva P., Corrado L., Natacci F., Castorina P., Wu B.L., Schneider G.H., Clementi M., Tenconi R., Korf B.R., Larizza L. (2000). NF1 Microdeletion Syndrome: Refined FISH Characterization of Sporadic and Familial Deletions with Locus-Specific Probes. Am. J. Hum. Genet..

[49] Prinja S., Gupta N., Verma R. (2010). Censoring in Clinical Trials: Review of Survival Analysis Techniques. Indian J. Community Med..

